# First Clinical Experience with a Novel 3D C-Arm-Based System for Navigated Percutaneous Thoracolumbar Pedicle Screw Placement

**DOI:** 10.3390/medicina58081111

**Published:** 2022-08-17

**Authors:** Eric Mandelka, Jula Gierse, Paul A. Gruetzner, Jochen Franke, Sven Y. Vetter

**Affiliations:** Department of Orthopedics and Trauma Surgery, BG Klinik Ludwigshafen, Ludwig-Guttmann-Str. 13, 67071 Ludwigshafen, Germany

**Keywords:** minimally invasive spine surgery, pedicle screw placement, accuracy, navigation, intraoperative imaging, radiation exposure, screw placement time

## Abstract

*Background and Objectives*: Navigated pedicle screw placement is becoming increasingly popular, as it has been shown to reduce the rate of screw misplacement. We present our intraoperative workflow and initial experience in terms of safety, efficiency, and clinical feasibility with a novel system for a 3D C-arm cone beam computed-tomography-based navigation of thoracolumbar pedicle screws. *Materials and Methods*: The first 20 consecutive cases of C-arm cone beam computed-tomography-based percutaneous pedicle screw placement using a novel navigation system were included in this study. Procedural data including screw placement time and patient radiation dose were prospectively collected. Final pedicle screw accuracy was assessed using the Gertzbein–Robbins grading system. *Results*: In total, 156 screws were placed. The screw accuracy was 94.9%. All the pedicle breaches occurred on the lateral pedicle wall, and none caused clinical complications. On average, a time of 2:42 min was required to place a screw. The mean intraoperative patient radiation exposure was 7.46 mSv. *Conclusions*: In summary, the investigated combination of C-arm CBCT-based navigation proved to be easy to implement and highly reliable. It facilitates the accurate and efficient percutaneous placement of pedicle screws in the thoracolumbar spine. The careful use of intraoperative imaging maintains the intraoperative radiation exposure to the patient at a moderate level.

## 1. Introduction

For numerous indications, dorsal stabilization with the placement of pedicle screws is a common treatment option [1,2,3,4,5]. However, this technique is associated with the risk of potentially severe complications due to neurovascular injury caused by misplaced screws [6]. In the past few years, the increasing use of minimally invasive spine surgery has resulted in a growing interest in navigation based on intraoperatively performed 3D imaging [7,8]. Several studies have reported a significant increase in the accuracy of screws placed with navigation compared to fluoroscopy-guided screw placement [9,10,11,12,13,14,15].

Different combinations of 3D-imaging devices and navigation systems for spinal navigation are available. Accordingly, numerous studies have been performed to investigate the image quality and radiation dose of the imaging devices as well as the accuracy and usability of navigation systems [16,17,18,19].

Since June 2021, a novel device enabling 3D C-arm CBCT-based navigation for the placement of thoracolumbar pedicle screws has been implemented. However, to date, no reports on its clinical use are available in the literature.

The aim of this study is to present our workflow for navigated minimally invasive pedicle screw placement, to report our initial experience in terms of clinical feasibility and efficiency, and to evaluate screw accuracy, patient radiation exposure, and the time needed for screw placement in the first clinical cases performed with the system.

## 2. Materials and Methods

The study was reviewed and approved by the ethics committee of the Medical Association Rhineland-Palatine, Germany (application number 2021-16061). All included patients gave verbal and written consent. All the procedures were in accordance with the ethical standards of the institutional and/or national research committee and with the 1964 Helsinki Declaration and its later amendments or comparable ethical standards.

The first 20 consecutive cases of percutaneous pedicle screw placement were performed by a single experienced spine surgeon (S.Y.V.) after implementation of the system in our institution. All patients had preoperative imaging with conventional anteroposterior and lateral X-rays as well as computed tomography (CT).

### 2.1. Intraoperative Workflow

Screw placement was performed using the navigation software of the Pulse platform (NuVasive Inc., San Diego, CA, USA). This device is composed of a monitor cart with two vertically stacked touch-sensitive flat screens and a mobile camera unit with two infrared cameras. Navigation was based on datasets acquired with a newest-generation mobile 3D C-arm CBCT (Cios Spin, Siemens Healthcare, Erlangen, Germany). To allow for referencing, two arrays were mounted on both sides of the flat panel detector of the C-arm.

**Figure 1:**
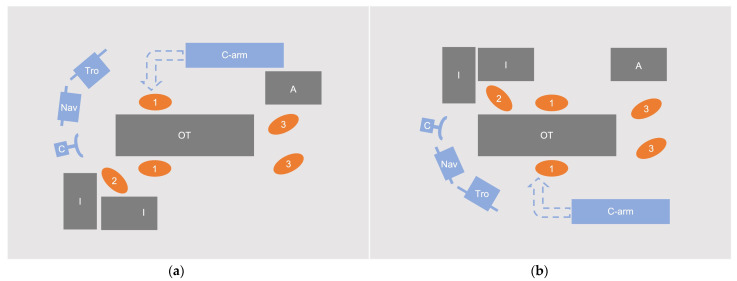
Different intraoperative settings for (**a**) upper thoracic procedures and (**b**) mid/lower thoracic and lumbosacral procedures. The system is placed at the end of the operating table for ergonomic viewing. 1: surgeons, 2: scrub nurse, 3: anesthesiologist/anesthesiologic nurse, A: anesthesiology unit, C: camera unit, C-arm: 3D C-arm, I: instrumentation trays, Nav: navigation monitor cart, OT: operating table, Tro: C-arm Trolley.

All the patients were placed on a radiolucent carbon-fiber surgical table in prone position. The orientation of the patient depended on the planned spinal levels for the instrumentation to avoid the interference of the C-arm with the table column and to account for the different curvature of the spine at upper thoracic and lumbar levels (Figure 1). In either setting, the camera of the navigation system is placed at the end of the operating table where the surgeon stands, and the monitor cart is flexibly positioned to ensure ergonomic viewing.

**Figure 2:**
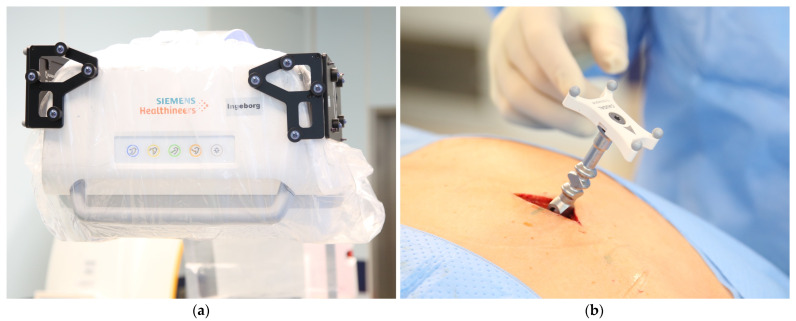
(**a**) Flat panel detector of the C-arm Cios Spin with sterile draping and referencing array. The spheres are only mounted on the side used for the procedure; (**b**) patient array attached to a spinous process close to the region of interest.

The correct spinal levels were identified using low-dose fluoroscopy before the preparation and draping of the patient. After a small midline incision and soft-tissue preparation, the patient reference array was mounted to a spinous process or the posterior iliac crest. For highest accuracy, it is recommended that a spinous process is chosen inside, or at least close to, the surgical field. Mounting on a fractured/instable spinous process should be avoided. The design and the comparatively small size of the array allowed for sufficient mobility of the instruments, even with the array mounted inside the region of interest (Figure 2).

After applying a sterile transparent cover to the C-arm, a registration scan was performed. Ventilation should be paused during scan acquisition to avoid motion artifacts and enhance both accuracy and image quality. While in most cases, the image quality in the low dose mode (200 images in 30 s) of the C-arm is highly sufficient for screw placement, to reduce artefacts, we recommend a referencing scan with 400 images for better image quality in obese patients or in patients with spinal implants.

After acquisition of the 3D scan, image data were automatically transferred to the navigation device via the NaviLink interface of the C-arm. After selection of the spine levels for instrumentation, screw placement was performed: First, navigation accuracy was verified with the navigated pointer on different anatomical landmarks. If the accuracy was adequate, the navigated drill guide was used to find the appropriate screw trajectory and make a small incision in the skin. Then, subcutaneous tissue and the fascia is performed. Of course, the technique presented here can also be used in open-approach surgery. To avoid soft-tissue traction on the instruments or the screw under insertion and the resulting effect on accuracy, blunt preparation of the trajectory down to the lamina vertebrae is recommended. Subsequently, the navigated drill guide was used to find the adequate trajectory with the help of the simultaneous visualization of the trajectory and the corresponding screw on the screen. Screw diameter and length can easily be adjusted on the touchscreen by OR personnel. After selecting the right screw size and diameter, electric drilling was performed with a 3.5 mm drill. The trajectory can be saved by clicking the corresponding field on the touchscreen, we recommend saving the trajectory during drilling. Using the drill guide, the drilling depth can be flexibly adjusted. From our experience, we recommend 30 mm for thoracolumbar screw placement, which is sufficient for drilling through the pedicle and beyond the posterior wall into the spongious bone of the vertebral body. The drill was removed with the drill guide held in place, and a flexible guide wire was inserted into the pedicle. Finally, the drill guide was removed entirely.

While some surgeons prefer to place every screw immediately after drilling, we recommend the drilling and placement of guide wires for all planned screws to avoid the mechanical manipulation of the spine, which may occur during screw insertion and might affect the navigation accuracy of the subsequent screws. Between the placement of different guide wires, accuracy should be checked using the navigated pointer.

On surgeon discretion, standard AP and lateral fluoroscopic views are performed to check guide-wire position. This allows potential misplacement to be detected before the screw is inserted, which is significantly thicker in diameter, and thus potentially more destructive. If guide-wire position cannot be evaluated or seems inadequate in standard fluoroscopic views, a control scan may be performed, followed by a correction of guide-wire position, if necessary.

**Figure 3:**
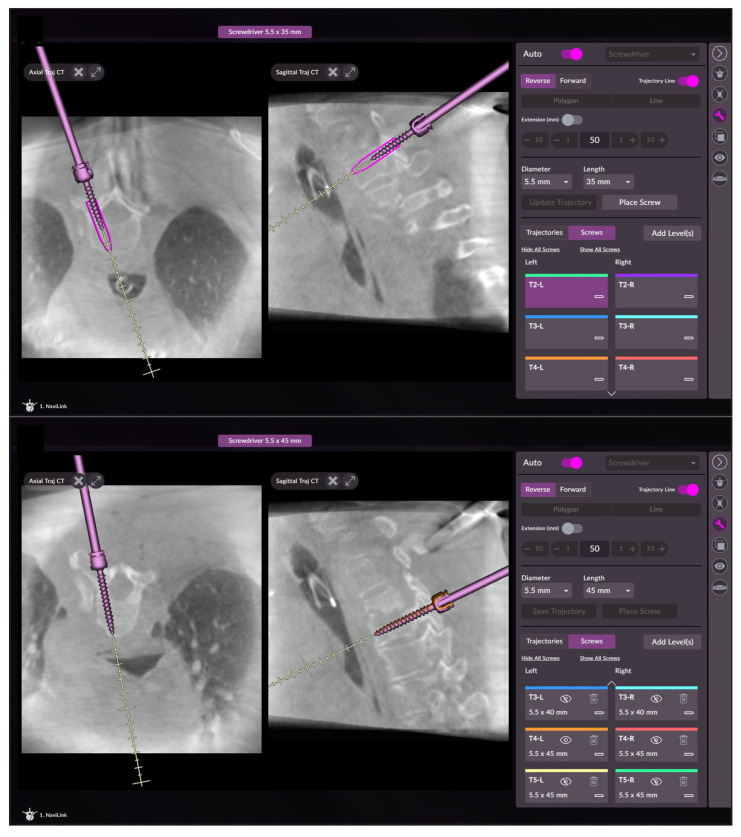
Screenshots of the navigation software during screw insertion with visualization of the planned position of the screw and with the correctly positioned screw.

The guide wire also facilitates locating the exact entry point for the cannulated screw (Reline MAS, NuVasive, San Diego, CA, USA), which is then inserted according to the drilled trajectory continuously visualized in axial and sagittal planes on the screen (Figure 3). After screw placement is completed, standard fluoroscopic views are performed. In certain cases, an intraoperative 3D scan to analyze final screw position may be performed, in order to allow intraoperative revision of misplaced screws.

### 2.2. Data Collection

The following parameters were prospectively collected:

1. Patient data including diagnosis, spinal levels to be instrumented, as well as the number of segments instrumented, and screws placed.
2. Procedural times during the procedures:
  - Operating time (OT, from first incision to suture);
  - Navigation time (NT, first insertion of the navigated pointer until after placement of the last screw);
  - Screw placement time (SPT) for every single screw (insertion of the drill guide until the removal of the screwdriver after screw placement).
3. Intraoperative patient radiation exposure as dose area product (DAP) and fluoroscopy time (FT) as documented in the imaging system dose report; for calculation of the effective dose (ED) in mSv, ED/DAP conversion factors of 0.29 (Body mass index, BMI < 25), 0.26 (BMI 25–30) and 0.23 (BMI > 30) were used [20].
4. Intraoperative revision of guide wires or screws.
5. Intraoperative complications or technical problems.
6. Postoperative complications (changes in neurovascular status, infection, wound healing disorder, revision surgery)
7. Pedicle screw accuracy was assessed in postoperative CT by an independent investigator with extensive experience according to Gertzbein–Robbins grading system with grades A (no pedicle breach) and B (pedicle breach < 2 mm) considered clinically acceptable and Grades C (pedicle breach < 4 mm), D (pedicle breach < 6 mm), and E (pedicle breach ≥ 6 mm) deemed unacceptable because of potential neurovascular injury [21].

### 2.3. Statistical Analysis

Statistical analysis was performed using Prism 8 (Graphpad Software, San Diego, CA, USA). Kolmogorov–Smirnov test was used to check for normal distribution of data. Descriptive statistics are shown as means and 95% confidence interval (95% CI) for continuous variables. Mann–Whitney U test was used to analyze the central tendencies of the differences. The significance level was set at *p* < 0.05.

## 3. Results

Overall, 156 screws were placed in 20 patients during 20 procedures.

### 3.1. Patient Data

Eight patients were female, and twelve patients were male. The mean age at the time of surgery was 68.6 years (95% CI 63.4–73.4 years), and mean BMI was 28.2 (95% CI 25.3–31.1). Eight procedures were performed at the thoracic level, four on the thoracolumbar junction, seven on the lumbar spine, and one on the lumbosacral junction. Fourteen procedures were performed for vertebra fractures and six for spondylodiscitis. The general patient data and procedure characteristics are displayed in Table 1.

### 3.2. Procedural Times

Overall, the mean operating time from the first incision to suture was 92.3 min (95% CI 82.6–102.0 min). For navigation alone, 35.7 min (95% CI 28.6–42.9 min) was required. The time needed to place every screw was also recorded. On average, a time of 2:42 min was required to perform wire and screw placement in a pedicle (95% CI 2:31–2:57 min).

### 3.3. Patient Radiation Exposure

The intraoperative dose area product, fluoroscopy time and calculated ED are shown in Table 2.

In addition to the registration scan, additional intraoperative 3D imaging was performed in 12 of 20 patients. Wire or screw position was checked in one and nine cases, respectively, while in two cases, a 3D scan was performed both after wire and screw placement (Case 4 and Case 10). As expected, DAP was significantly higher in cases with a control scan compared with cases without additional intraoperative 3D imaging (*p* = 0.001).

### 3.4. Intraoperative Revisions and Intra-/Postoperative Complications

Intraoperative screw revision after 3D imaging was performed in two cases (one screw each, 1.3% of all screws). No intra- or postoperative complications or technical malfunctions were reported. Revision surgery was not performed in any cases.

### 3.5. Accuracy

**Figure 4:**
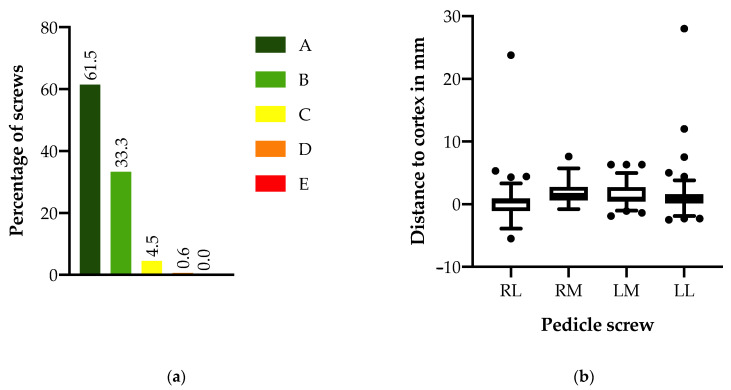
Screw accuracy: (**a**) percentage of screws according to different Gertzbein–Robbins system (GRS) grades. A: GRS grade A, B: GRS grade B, C: GRS grade C, D: GRS grade D, E: GRS grade E. (**b**) distance between the pedicle screws and the medial/lateral cortices with the area marked in red depicting perforations ≥ 2 mm. RL: right lateral, RM: right medial, LM: left medial, LL: left lateral.

The final screw accuracy was 94.9% with 96 screws graded as A, 52 graded as B, 7 graded as C, and 1 graded as D using Gertzbein–Robbins grading system (Figure 4). All eight screws graded as C or D showed a pedicle breach on the lateral side. Out of the eight screws, five showed an in–out–in configuration, while one screw also showed a perforation of the lateral vertebral wall. In the remaining two cases, the screw diameter was larger than the diameter of the pedicle (measured in preoperative CT), resulting in an in–out–in configuration of the screws.

## 4. Discussion

The purpose of this study was to present our intraoperative workflow for percutaneous pedicle screw placement using a novel navigation system and to report our initial experience concerning its feasibility and reliability, as well as the results of the first performed clinical cases. To the best of our knowledge, there are currently no other studies on the in-patient use and evaluation of this system.

The first 20 patients in which minimally invasive screw placement was performed were included in the study after implementation of the system. Due to our institution’s focus on trauma cases, most procedures were performed on patients with vertebral fractures. Since both the performing surgeon and the OR staff already had experience with navigation in general, particularly the navigation of pedicle screws in the cervical spine, the presented workflow could be established within a very short time. The efficiency of the workflow is confirmed by an average screw placement time of 2:42 min, which is a competitive time compared with other studies [22,23,24].

There is extensive evidence in the literature that navigated procedures achieve a higher accuracy of screw placement in the pedicle compared to freehand and fluoroscopically assisted procedures [9,10,11,12,13,14,15]. On the other hand, studies remain inconclusive as to whether this is achieved at the expense of a longer operative time and increased radiation exposure for the patient, which is a frequent criticism from those who oppose the increased use of spinal navigation [25,26]. Nevertheless, in their study, Tkatschenko et al. concluded that the benefits of 3D navigation in terms of higher accuracy and reduced revision rates outweigh the potential one-time increase in radiation exposure. The reduced exposure for surgeons and operating room staff may be even more significant since they are exposed to a considerable amount of radiation during their working life, especially in spine surgery [27,28,29]. In a study by Villard et al. the use of navigation reduced the surgeon’s radiation exposure by a factor of 10 compared with fluoroscopy-assisted pedicle screw placement [30]. This is due to the fact that navigation minimizes the need to use fluoroscopy while the surgeon and operating room personnel leave the controlled area during scan acquisition, leaving only the patient exposed.

Although no comparison was made with conventional screw placement in this study, the average effective dose to the patient of 7.46 mSv for the included procedures appears to be reasonable compared with data in the literature [31,32].

In their experimental study, Foster et al. investigated the effective dose for different imaging devices including the C-arm used in the present study. In a human specimen with pedicle screws inserted into the lumbar spine, they reported an effective dose of 1.9 mSv, 4.0 mSv, and 4.6 mSv for a single 3D scan with low-, medium-, and high-dose protocols, respectively, using similar ED/DAP conversion factors [33].

Keil et al. were able to show in their study that the C-arm CBCT used in this study provides sufficient image quality to evaluate the relevant structures with regard to pedicle screw placement [34]. In the present study, the intraoperative 3D control of the wire and/or screw position was performed in 12 of 20 patients, potentially eliminating the need for postoperative standard-dose CT with an estimated effective dose of approximately 5 to 10 mSv [26,32,35].

In two cases, the revision of an inserted screw was necessary following intraoperative 3D imaging. An evaluation by an independent investigator resulted in a final screw accuracy of 94.9%. In 8 of 156 screws, a potentially relevant perforation of the pedicle was present, which occurred on the lateral side in all cases. In seven out of eight cases, these screws showed an in–out–in configuration and/or were larger in diameter than the pedicle itself. Pedicle screws placed in an in–out–in configuration have been described to be biomechanically superior compared to a fully intrapedicular position [36]. In addition, Burström et al. demonstrated that the achieved accuracy can vary significantly depending on the size of the screws used [37]. Complications of these pedicle perforations did not occur in any of the cases.

Our results are in accordance with other studies investigating the accuracy of navigated pedicle screw placement. In the study by Tkatschenko mentioned above, an accuracy of 95.8% was reached with percutaneous C-arm-based navigation, which also used the Gertzbein–Robbins classification with a cut-off value of 2 mm [27]. In their meta-analysis, Perdomo-Pantoja et al. investigated the accuracy rates of different screw placement techniques and reported similar results for navigated procedures (95.5%) [10].

In a recently published experimental study, the combination of a C-arm CBCT and navigation system reached an accuracy rate of 97.5%, which was non-inferior to iCT navigation and superior to O-arm CBCT-based navigation [38]. Whether the results obtained in this experimental setting can also be achieved in everyday clinical practice will need to be investigated in future studies.

As with all navigated procedures, potential pitfalls must be considered. The presumed constant visualization of anatomy and implants can provide a deceptive sense of security. Therefore, regular verification using anatomical landmarks must be performed to detect potential navigation inaccuracies [39,40].

Compared with ‘mobile’ iCT, which has been frequently investigated in the literature, the C-arm CBCT can be more easily relocated [34]. However, this comes with the risk of mechanical impact during transfers, for example, between different operating rooms, which can have potentially devastating consequences for the accuracy of navigation.

Overall, this study has several limitations:

First, we present an observational case series with a limited number of cases. Nevertheless, this study is the first description of the clinical application of this system under investigation. The aim of this study was not to test the accuracy on a large number of screws, but to describe the feasibility and reliability, as well as a highly efficient intraoperative workflow from which other users may benefit.

Second, because the first clinical cases were included, we did not consider a potential learning curve with the new system; however, this would be more likely in an inexperienced surgeon [41].

Because regular navigated pedicle screw placement was not performed in the thoracolumbar spine prior to the implementation of the system, it was not possible to create a control group based on conventional procedures performed by the same surgeon.

Further studies should aim to investigate relevant parameters—such as accuracy, radiation exposure, and time required—using larger patient populations, comparing these parameters with fluoroscopically assisted pedicle screw placement and using other combinations of imaging devices and navigation systems.

## 5. Conclusions

In summary, the investigated combination of C-arm CBCT-based navigation proved to be easy to implement and highly reliable. This facilitates the accurate and efficient percutaneous placement of pedicle screws in the thoracolumbar spine. The careful use of intraoperative imaging maintains the intraoperative radiation exposure for the patient at a moderate level.

## Figures and Tables

**Table 1:** General patient data and procedure characteristics.

Case	Age	Diagnosis	No. of Screws Placed	No. of Levels Fused	Levels Fused	Further Interventions	OT ^1^ [min]	NT ^2^ [min]	mSPT ^3^ [mm:ss]
1	78	Spondylodiscitis	8	3	L3-S1	None	86	54	3:18
2	73	Fracture T12/L1	8	3	T11-L2	None	94	43	3:27
3	65	Fracture L2	6	2	T12-L2	None	74	31	2:02
4	82	Fracture L2/3	8	3	L1-L4	None	116	66	5:22
5	57	Fracture T8/9	8	3	T7-T10	None	90	41	2:45
6	78	Fracture T3	10	4	T1-T5	None	107	58	3:17
7	54	Fracture T4	8	3	T2-T5	None	89	34	2:21
8	78	Spondylodiscitis	8	3	L2-L5	None	77	46	1:39
9	69	Fracture L2	4	2	L1 onto L3	BSC * L2	58	18	2:57
10	80	Fracture T7	8	3	T5-T8	None	93	63	4:07
11	82	Spondylodiscitis	8	3	T7-T10	None	95	54	2:34
12	78	Fracture L1	8	4	T11/12 onto L2/3	CA#	130	32	1:56
13	85	Fracture L4	8	3	L2-L5	None	132	43	3:16
14	71	Fracture T12	8	4	T10/11 onto L1/2	CA#	82	26	1:31
15	58	Fracture L2	6	2	L1-L3	None	62	24	2:08
16	59	Spondylodiscitis	8	3	T2-T5	None	100	47	3:09
17	61	Fracture T6	8	4	T4/5 onto T7/8	BSC * T6, CA #	106	42	1:53
18	56	Spondylodiscitis	12	5	L1/2/3 onto L4/5/S1	None	112	52	2:11
19	58	Fracture L2	6	3	L1-L3	None	69	28	2:45
20	50	Spondylodiscitis	8	3	T6-T9	None	73	33	1:48

^1^ OT: operating time; ^2^ NT: navigation time; ^3^ mSPT: mean screw placement time; * BSC: bone sample collection; # CA: cement augmentation of screws.

**Table 2:** Intraoperative patient radiation exposure.

	Mean	95% CI ^1^
Dose area product (Gycm^2^)	29.78	22.43–37.12
Fluoroscopy time (s)	93.7	80.6–106.8
Effective dose (mSv)	7.46	5.77–9.14

^1^ 95% CI: 95% confidence interval.

## Data Availability

All the data and statistics are available on reasonable request from the corresponding author.
